# Fingerprinting antioxidative activities in plants

**DOI:** 10.1186/1746-4811-5-2

**Published:** 2009-01-26

**Authors:** Livia Saleh, Christoph Plieth

**Affiliations:** 1 Zentrum für Biochemie und Molekularbiologie, Universität Kiel, Am Botanischen Garten 9, 24118 Kiel, Germany

## Abstract

**Background:**

A plethora of concurrent cellular activities is mobilised in the adaptation of plants to adverse environmental conditions. This response can be quantified by physiological experiments or metabolic profiling. The intention of this work is to reduce the number of metabolic processes studied to a minimum of relevant parameters with a maximum yield of information. Therefore, we inspected 'summary parameters' characteristic for whole classes of antioxidative metabolites and key enzymes.

**Results:**

Three bioluminescence assays are presented. A horseradish peroxidase-based total antioxidative capacity (TAC) assay is used to probe low molecular weight antioxidants. Peroxidases are quantified by their luminol converting activity (LUPO). Finally, we quantify high molecular weight superoxide anion scavenging activity (SOSA) using coelenterazine.

Experiments with *Lepidium sativum *L. show how salt, drought, cold, and heat influence the antioxidative system represented here by TAC, LUPO, SOSA, catalase, and glutathione reductase (GR). LUPO and SOSA run anti-parallel under all investigated stress conditions suggesting shifts in antioxidative functions rather than formation of antioxidative power. TAC runs in parallel with GR. This indicates that a majority of low molecular weight antioxidants in plants is represented by glutathione.

**Conclusion:**

The set of assays presented here is capable of characterising antioxidative activities in plants. It is inexpensive, quick and reproducible and delivers quantitative data. 'Summary parameters' like TAC, LUPO, and SOSA are quantitative traits which may be promising for implementation in high-throughput screening for robustness of novel mutants, transgenics, or breeds.

## Background

### Plant stress response, oxidative burst and the antioxidative systems

Changes in the environment may represent so-called 'stress situations' in plants. 'Stress' is typically characterised by excessive formation and release of reactive oxygen species (ROS; e.g. superoxide anion O_2_^-·^, hydrogen peroxide H_2_O_2_, singulet oxygen O^·^, and hydroxyl radical OH^·^) and consequently causes changes in the redox environment on cellular level [1]. These phenomena are summarised with the term 'oxidative burst' [2]. An oxidative burst is followed by multiple responses in transcription [3,4], translation [5,6], protein activity [7,8], metabolism and possibly programmed cell death [9,10].

ROS formation always occurs during normal growth and development, particularly in sub-cellular locations with high enzymatic redox turnover. Thus, the formation and destruction of ROS are well balanced in plant cells and under the control of a complex antioxidative system [11,12]. The antioxidative system mainly consists of antioxidative enzymes (e.g. APX, GPX, SOD, CAT, GR) catalysing electron transfer to ROS using low molecular weight antioxidants (e.g. ascorbate, tocopherol, GSH) as electron and proton donors [13,14].

However, during an 'oxidative burst' this equilibrium becomes unbalanced and the organism is forced to adjust its antioxidative system in order to cope better with the current or a future stress situation. A pre-requisite for this is a signalling network that is able to switch antioxidative and metabolic redox capacities. Consequently, ROS are also considered to be signalling components which specifically trigger antioxidative responses [15-19]. Many studies show how and where the formation of ROS occurs (e.g. for review see [20]). Although particular effects on the antioxidative system are well characterised, little is known about general metabolic effects.

### Probing the antioxidative system in plants

Transcript profiling and proteomic approaches are used for tracking adaptations in antioxidative system in response to environmental changes. However, for proof of functional changes a third trace of evidence using metabolic profiling has to be adopted [21-23]. Here, we present a first approach to quantify changes in the antioxidative system of plants after abiotic stress challenge using 'summary parameters'. To achieve this, we have optimised widely used chemiluminescence assays.

These assays allow the quantification of:

1) the total antioxidative capacity (TAC) of low molecular weight metabolites,

2) the luminol converting peroxidase activity (LUPO) in plant tissue,

3) the total superoxide scavenging activity (SOSA) of high molecular weight compounds including SODs.

All assays yield information about the general antioxidative status and about specific aspects of the antioxidative system, rather than exact data for single antioxidant species. For each luminometric assay information is given on how to tune the sensitivity and how to fit assay performance to the requirements of the biological specimen under study.

We compare results obtained from probing *Lepidium sativum *under different abiotic stress situations and show that common photometric assays for catalase (CAT) and glutathione reductase (GR) activities can easily be run in parallel.

### The HRP-catalysed luminol-reaction as a tool to estimate total antioxidative capacity (TAC)

Antioxidative power is generally defined as the ability to scavenge ROS and antioxidative capacity is expressed in terms of concentration of a pure antioxidative substance. TAC can thus be used as a marker to detect changes of antioxidative metabolism during oxidative stress.

**Figure 1:**
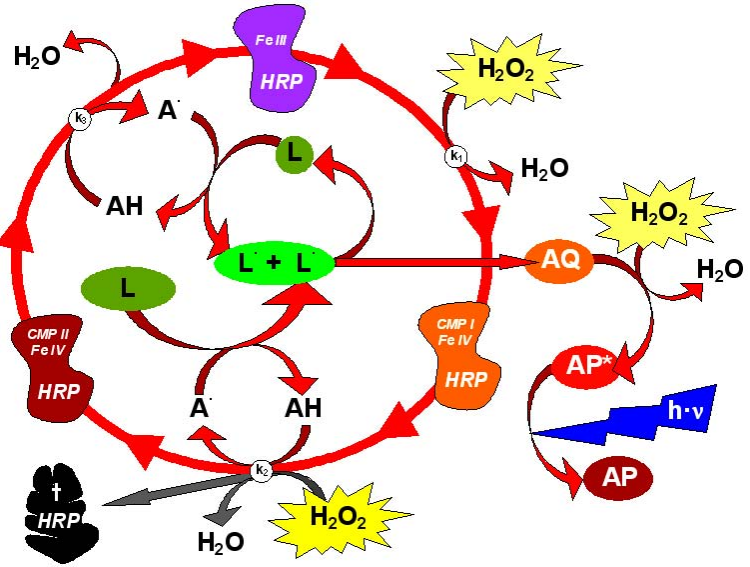
**The enhanced catalytic horseradish peroxidase cycle (adapted from **[25]**)**. Luminol (L) is used as substrate for the light generating process. A di-aza-quinone (AQ) is formed as intermediate. This in turn is oxidised by hydrogen peroxide (H_2_O_2_) to form an excited state of aminophtalate (AP*). The final step is the emission of blue (420 nm) light (***h·ν***) when the excited AP* returns to its ground state. Luminol can work as a substrate of the horseradish peroxidase (HRP). However, for analytical purposes an intermediate aromatic hydrogen donor (AH) is added. This enhancer serves as primary substrate for the HRP and its radical (A^·^) subducts electrons from luminol (L) and thus forms the radical form L^·^. AQ is formed by electron transfer between two L^·^. The HRP compound I-state (CMP I) is sensitive to excess of H_2_O_2 _[33] and can undergo a peroxide inactivation (so-called 'suicide reaction'; grey arrows) when the concentration of H_2_O_2 _added to start the light emitting reaction is too high (compare Fig. 1.4 in additional file 1).

The general basis of TAC assays is a redox reaction driven by a ROS (typically H_2_O_2_). The signal output produced, is subsequently quenched by the addition of a sample with ROS-scavenging properties. Luminol (L = 3-Aminophthalhydrazide) is a frequently used reagent which emits light when oxidised by H_2_O_2 _to aminophtalate (AP) under alkaline conditions (see Fig. 1.1 in additional file 1). This chemiluminescence reaction can be catalysed by peroxidases (POs) such as horseradish peroxidase (HRP). HRP is a widely used enzyme that oxidises phenolic compounds with hydrogen peroxide (H_2_O_2_) as oxidant [24]. The HRP-catalysed luminol reaction involves three steps by which the HRP protein undergoes conformational changes until functional HRP is regenerated (Figure 1, [25,26]).

Molecular oxygen (O_2_) is not involved in this reaction [27,28] as long as there is a reactant [29]. However, other additives are required for the HRP reaction that either serve as primary electron donors or as excipients that enhance enzyme turnover and stability. Calcium is an essential cofactor for HRP [25,30]. Phenolic electron donators with higher affinity to HRP than luminol (e.g. iodophenol) enhance the luminescence drastically [31]. Also a surfactant is beneficial as it stabilises the protein, and enhances the light output ([32,33], Fig. 1.2 in additional file 1).

The pH optimum of the reaction is around pH 8.7 (Fig. 1.3 in additional file 1). Although the light reaction is driven by H_2_O_2_, an excess of it inactivates HRP ([26,33], Fig. 1.4 in additional file 1).

**Figure 2:**
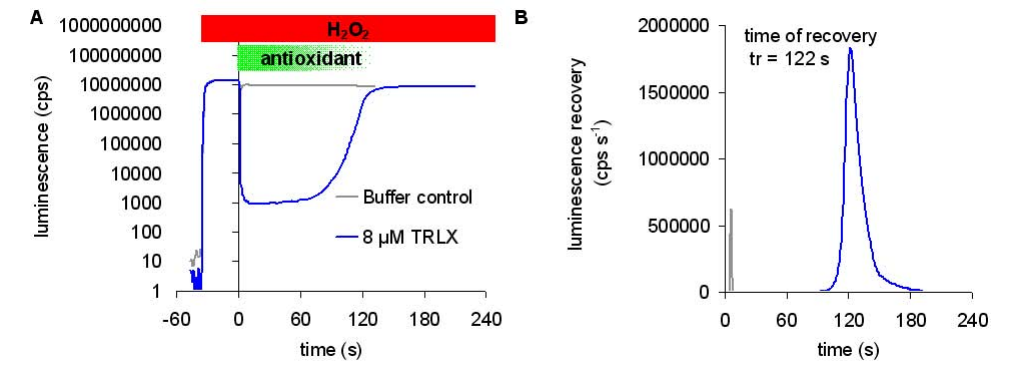
**Quenching of HRP-generated light output by an antioxidant**. **A: **Light generation of the HRP-catalysed luminol reaction was triggered by the addition of H_2_O_2 _(at time t_0 _= -36 s). The reaction is quenched by the addition of an antioxidant when a constant output signal is established. Here Trolox™ ((±)-6-Hydroxy-2,5,7,8-tetramethylchromane-2-carboxylic acid) a water soluble derivative of tocopherol (vitamin E) was used. The antioxidant is oxidised by H_2_O_2 _thus inhibiting the HRP-catalysed reaction. When the antioxidant is depleted, the HRP reaction resumes and the light signal recovers. **B**: The time point of signal recovery t_r _depends on the amount of added antioxidant and is defined here as the time where the rate of signal increase is maximum. The curves here are derived from the curves in A and represent the first derivative of the running mean (n = 10) on the raw data. Luminescence is given here in counts per second (cps) and its recovery in cps per s.

The reaction is started by the addition of H_2_O_2 _(details in Methods section). When a constant light output is recorded, an antioxidant containing sample is added (Figure 2). The luminol-HRP electron transfer is then inhibited due to the competition of the antioxidants with luminol.

Light output is quenched according to the amount of antioxidant. When the antioxidative capacity (AC) of the added sample is exhausted, the HRP-catalysed electron transfer from luminol continues and light output recovers. The amount of quenching (see Eq.1 additional file 1) and the time required for signal recovery after sample addition (i.e. the time point with maximal signal increase after oxidation of the sample, Figure 2B) are dependent on the amount of antioxidant added. The recovery times of specific amounts of a pure antioxidant are used to calibrate the assay (see Fig. 1.5 in additional file 1).

For each sample to be quantified the recovery time is measured and the result is given in terms of molar concentration of the pure antioxidant used for calibration. The result has to be related to fresh weight, dry weight, or total protein content of the sample.

HRP is employed in a wide range of analytical assays [34]. In particular, the HRP-based TAC assay is used in medical research and food science, mainly to probe the TAC of blood serum [35], beverages [36], vegetables, and other foods [37].

Tissue samples from plant material always contain the full complement of their antioxidative factors. This includes peroxidases and catalases which may compete with HRP in the assay and thus influence the light output and lead to erroneous TAC values. Hence, the low molecular weight antioxidants to be quantified have to be separated from enzymatic activities. Therefore membrane filtration with MWCO of 10 kDa has been found to be useful.

### The LUPO-assay: Probing peroxidase activity with luminol

Peroxidases are widely distributed among living organisms and have many physiological functions [14,38]. They exhibit a broad substrate specificity and require organic substrates for catalysis [39]. Many other peroxidases besides HRP also accept luminol as electron donor (L, see Fig. 2.1 in additional file 2). Luminol converting peroxidases (LUPO) can readily be quantified by their light yield (Fig. 2.2, 2.3, 2.4 in additional file 2). Since low molecular weight H_2_O_2_-scavengers may interfere with the LUPO reaction, the sample has to be dialysed before use (details in Methods section). The recorded light output is linearly correlated to the amount of peroxidase and, if required, can be expressed in equivalents of a standard peroxidase (Fig. 2.4A in additional file 2).

### The SOSA-assay: Probing superoxide scavengers with coelenterazine

The superoxide anion (O_2_^-·^) is formed by single electron transfer from over-reduced redox enzymes to molecular oxygen. It has a short lifetime in living cells [40] and is disproportionated to H_2_O_2 _and molecular oxygen. Plants possess several superoxide dismutases (SODs) scavenging superoxide anions enzymatically [41-43]. Additionally, non-enzymatic O_2_^-·^-scavengers are also found in plants [44].

Like the TAC assay, the method for assaying the superoxide anion scavenging activity (SOSA) presented here, is based on the quenching of chemiluminescence. Here, the light-yielding substrate is coelenterazine (CTZ), a specific O_2_^-·^-indicator ([45,46], Fig. 3.1 in additional file 3).

**Figure 3:**
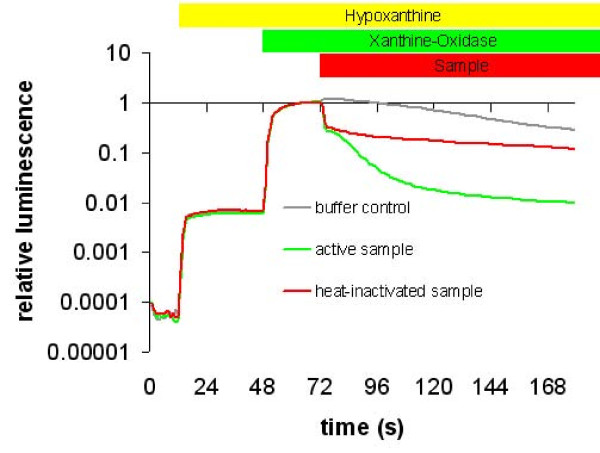
**Quenching of CTZ chemiluminescence by superoxide scavengers from *Lepidium***. CTZ was mixed with hypoxanthine at t = 12 s. This gave a background luminescence due to the presence of ambient oxygen. The superoxide yielding reaction was started by injection of xanthine oxidase to the assay mix at t = 48 s. Light output was quenched by an extract from *Lepidium *containing superoxide scavengers at t = 72 s (green line). The red line represents non-enzymatic scavenging of a heat-inactivated (30 min at 95°C) sample. The grey line is the control experiment with buffer injected. The steady state luminescence after starting the reaction with XOD (62 s < t < 72 s) was used to normalise the data.

Due to its very short lifetime, O_2_^-· ^has to be generated *in situ*. The most convenient method to generate O_2_^-· ^employs xanthine oxidase (XOD) [47]. While XOD is generating O_2_^-· ^(Fig. 3.1A in additional file 3) and thus producing light in presence of CTZ (Fig. 3.1B in additional file 3), any O_2_^-·^-scavenger in the assay reduces the steady state O_2_^-· ^concentration and thus light output. This quenching of CTZ luminescence is used to quantify the scavenger activity. In the dialysed sample non-enzymatic high molecular weight scavengers [48] can be distinguished from enzymatic (i.e. SOD) scavenging activities (Figure 3) (also see Fig. 3.2 in additional file 3).

For a SOSA assay, several factors have to be considered:

1) The XOD needs a physiological pH and calcium buffering for high superoxide anion yield. From all available XODs the one from bovine milk does produce sufficient O_2_^-· ^[49].

2) There are many CTZ analogues available for use as O_2_^-· ^indicators (Tab. 1 in additional file 3). Each produces different luminescence yield (Fig. 3.3A, B in additional file 3). However, the important feature for the assay is the signal-to-background luminescence ratio ([50], Fig. 3.3C in additional file 3). CTZ needs careful handling since it is easily oxidised by ambient oxygen when in solution (details provided in the Methods section and in four additional files). The CTZ concentration determines the duration of constant light output (Fig. 3.4 additional file 3).

3) A surfactant is needed to maximize luminescence yield.

## Results and discussion

**Figure 4:**
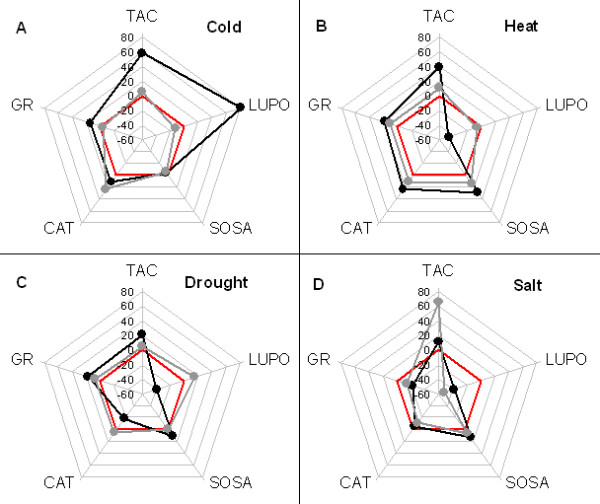
**Fingerprints of antioxidative activities in *Lepidium sativum *after abiotic stress**. The percentage change in the five screened parameters is represented by the five radial axes (data from Figures 5F, 6F, 7F, and 8F). The red pentagon in each panel represents the line of no change. Black polygons represent the antioxidative status immediately after stress treatment. Grey polygons represent the status after stress recovery. Each point is average of 5 technical replicates run on biological material pooled from 3 independent experiments. Standard deviations are given in Figures 5F–8F.

*Lepidium sativum *plants were challenged with four different types of abiotic stress. After stress and a recovery period (see Figs. 4.1 in additional file 4) plant material was processed (see Fig. 4.2 in additional file 4) and the antioxidative status was fingerprinted. Changes of screening parameters were drawn as polygon for each sample (Figure 4). The area of each polygon then gives a quantitative impression of changes in total antioxidative power of the sample.

### Cold Stress: Is adaptation governed by posttranslational events?

Adaptation to cold and formation of freezing tolerance involves a cascade of cellular events beginning with Ca^2+^- and IP_3_-mediated signal transduction, transcription factor activation and gene induction [51-54], leading to metabolic changes. Cold-induced metabolic changes have been studied and many metabolites and enzymes involved in cold adaptation have been identified [21,54-56]. However, the whole activation cascade is expected to work very slowly at 0°C. Thus, it seems that other posttranslational events are involved. This has been shown in particular for GR. Here [57], an increase in substrate affinity after cold treatment is reported rather than an increase in enzyme level or in GR transcripts.

We used five day old garden cress (*Lepidium sativum*) seedlings and applied a cold period (0°C) over night (12 h) and allowed recovery (12 h) under normal growth conditions thereafter.

**Figure 5:**
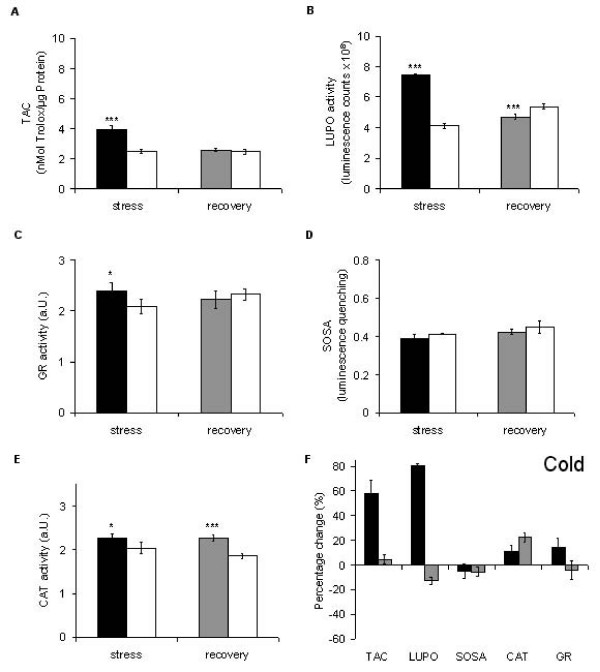
**Cold-induced alterations in the antioxidative system of *Lepidium sativum***. For each of the five screened parameter (TAC, LUPO, SOSA, CAT, GR) the status after stress treatment (12 h at 0°C) is given (black bars) and compared to the control of untreated plants (white bars). The grey bars represent the status of the particular parameter after 12 h recovery. For TAC (A) the recovery time in the luminescence signal was calibrated in terms of Trolox equivalents related to the protein content of the sample. The percentage change for each parameter is summarised (F) and significance according to Student's T-Test is marked with asterisks (* p < 0.05; ** p < 0.01; *** p < 0.001). Each column represents the average of five technical replicates run on pooled plant material from three independent growth and treatment experiments. Error bars represent StDv.

During a 12 h cold period, TAC is increased (Fig. 5A) and enzymatic activities (particularly peroxidases) are activated (Figure 5B). This reverts back to normal level during recovery. In contrast, the superoxide anion scavenging activity (SOSA) appears to be unaltered (Figure 5D). The enzyme activities (LUPO, GR and CAT) are also up-regulated during the cold period (Figures 5B, C, E). Most prominent is LUPO activity which increases by about 80% compared to control plants. CAT up-regulation seems to continue during the recovery period, while GR is regulated back to normal and LUPO is even down-regulated below normal. These results are in line with previous reports [58,59] which also showed induction of CAT and PO on the metabolic and transcriptional level during the first 12 h of cold.

When comparing the polygons in Figure 4, it becomes obvious that cold treatment (Figure 4A) makes a higher impact than all other stress factors (Figures 4B–D). This has already been noticed by others [21]. For many physiological responses the rate of temperature change (i.e. cooling rate) and not the steady state temperature is of importance ([60], for review see [61]). This has in particular been shown for Ca^2+ ^signalling [52,62]. Thus, it would be no surprise if this was also the case for the antioxidative parameters studied here. The substantial increase in the antioxidants and peroxidases during cold treatment (Figures 5A, B) is probably due to the cold shock applied. A more moderate treatment (slow cooling) may have less impact on the antioxidative system.

### Heat stress: Increase in metabolism brings about an increase in superoxide formation

Heat directly leads to oxidative damage. Cellular protection mechanisms against this involve different molecular cues. Up-regulation of genes and expression of heat-shock proteins (HSP) are well established responses to heat stress in plants [63,64].

For heat treatment we challenged *Lepidium sativum *seedlings for 6 h with 42°C. We chose a dark incubator to avoid light-induced additive effects which have been reported earlier [64]. The treatment was started in the morning and the plants were harvested about midday. For recovery a period of 6 h under normal growth conditions was allowed.

**Figure 6:**
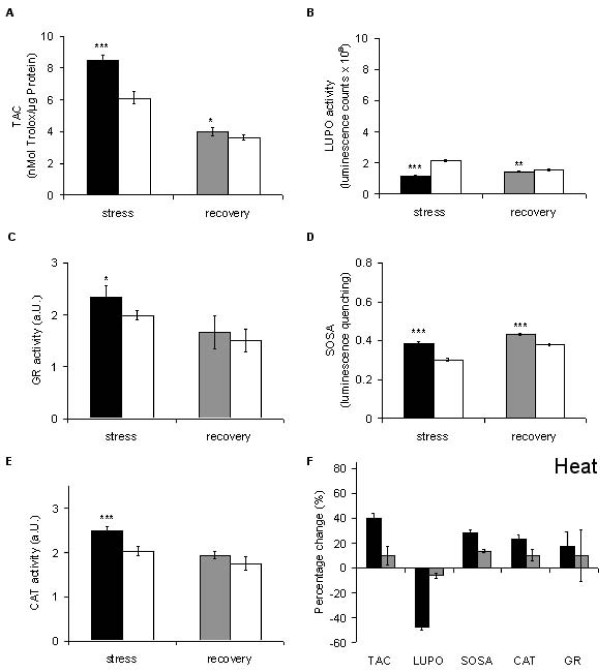
**Heat-induced alterations in the antioxidative system of *Lepidium sativum***. TAC, LUPO, SOSA, CAT, GR were measured after heat treatment (6 h at 42°C; black bars) and compared to the control of untreated plants (white bars). The grey bars represent the status of the particular parameter after 6 h recovery. Data presentation as in Figure 5.

The fingerprint (Figure 4B) reveals a mobilisation of TAC, SOSA, CAT, and GR. Nevertheless, there is no significant change in the area of the black polygon caused by massive reduction of LUPO (Figure 6B). The first prominent finding, up-regulation of SOSA (Figure 6D), implies that there is apparently a demand for O_2_^- ^scavengers, suggesting excessive formation of superoxide during heat treatment. Thus, in order to cope with high concentrations of hydrogen peroxide generated during superoxide dismutation, the plant seems to mobilise low molecular weight antioxidants and catalases as well (Figures 6A, E). The second finding is a massive down-regulation of LUPO (Figure 6B). It returns to normal level when the plant is allowed to recover from heat treatment. In accordance with our findings, are results obtained by heat treatment of *Arabidopsis *plants. Panchuk *et.al*. [65] found, that the total activity of ascorbate peroxidase in *Arabidopsis *was strongly reduced after several hours exposure to 44°C. Although peroxidases are generally believed to be heat stable, it has been shown that they suffer even from moderately raised temperature (42°C) [66]. In order to prove that this is also the case for the LUPO activity in *Lepidium *we performed a control experiment (Fig. 2.5 in additional file 2). The results show that LUPO is heat sensitive. Thus the reduction in LUPO (Figure 6B) is probably due to direct heat inactivation.

### Salt stress: A 'two-in-one' abiotic challenge

Different mechanisms are involved when plants respond to salt stress, namely defence against the osmotic effect of high salinity and against the ion toxicity. Apart from proline [67,68] no specific profiling of antioxidative metabolites has to our knowledge been reported for salt stress.

For salt stress treatment in this study, hydroponically grown *Lepidium sativum *seedlings were exposed to high salt concentrations (150 mM NaCl) for 24 hours. Afterwards a 24 h recovery period in 0.5 × MS medium was applied.

**Figure 7:**
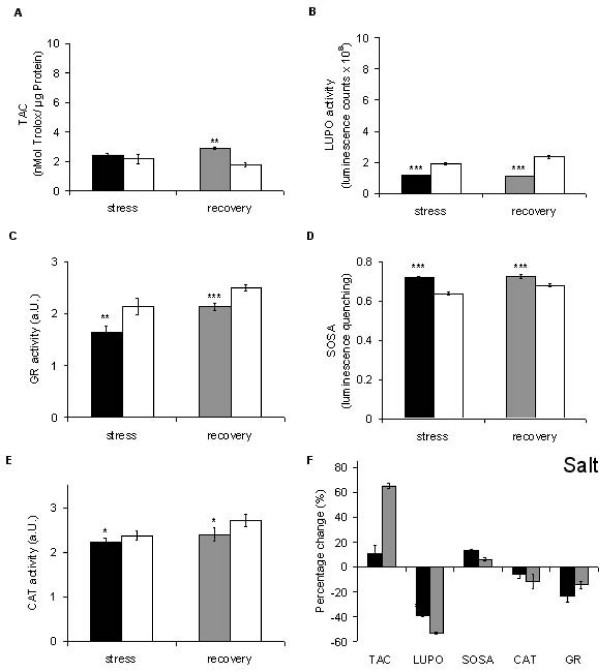
**Salt-induced alterations in the antioxidative system of *Lepidium sativum***. Salt treatment (24 h at 150 mM NaCl in 0.5 × MS medium; black bars) is compared to the untreated plants (white bars). The grey bars represent the status after 24 h recovery. Data presentation as in Figure 5.

There are numerous studies where salt stress induced responses of antioxidative enzymes were investigated. On the one hand, our results backup previous findings [69-71], since they show that SOSA is up-regulated during salt stress (Figure 4D). On the other hand, our results contrast with other studies. Peroxidase activity, for instance, increased significantly under salt stress in *Trigonella *species [72]. This inconsistency (Figure 7B) can be explained by the different conditions and time scales used for challenging plants with abiotic stress. Other previous work revealed that plants having a higher capability to neutralize ROS also have a higher salt tolerance [73]. In particular, Demiral and Türkan [74] demonstrated that a salt-tolerant rice cultivar (Pokkali) up-regulated CAT and APX activities during stress, whereas the salt-sensitive IR-28 does not exhibit this response. Gosset *et al*. [75] reported a significant increase in the activity of antioxidative enzymes in the salt-tolerant cultivar of cotton during salt stress. These results also indicate that fingerprinting the antioxidative system may allow the distinction of tolerant from non-tolerant lines. Here, salt stress does not influence TAC (Figure 7A), but inhibits GR and CAT (Figures 7C, E).

After recovery the salt stress response fingerprint does not return to normal (Figure 4D, grey polygon), as seen with all other abiotic treatments (Figures 4A–C). This is because the 'recovery treatment' (exchange of salt solution by normal nutrition medium) implies an additional challenge, (hypoosmotic stress), which provokes another shift of the antioxidative system. TAC is restored and even significantly up-regulated (Figure 7A and Figure 4D). Here, it cannot be distinguished whether this is an effect of the concurrent hypoosmotic shock or simply a relief of the 'high ion' effect on the involved enzymes.

### Drought stress: A matter of NADPH reducing power or of cellular ionic milieu?

Drought stress (i.e. water deficiency) generally depends on osmotic pressure changes. Since 'drought' cannot be precisely adjusted like temperature or salt concentration, in many previous studies osmotic stress produced by mannitol solutions was taken as a surrogate for drought [76].

Here, we avoided mannitol treatment since it is known to be a ROS scavenger [77], which would interfere with any antioxidative fingerprint experiments. We therefore applied a 24 h drought period by total withdrawal of nutrient medium from hydroponically grown *Lepidium *seedlings. Withered plants completely regained turgor during the following recovery period.

**Figure 8:**
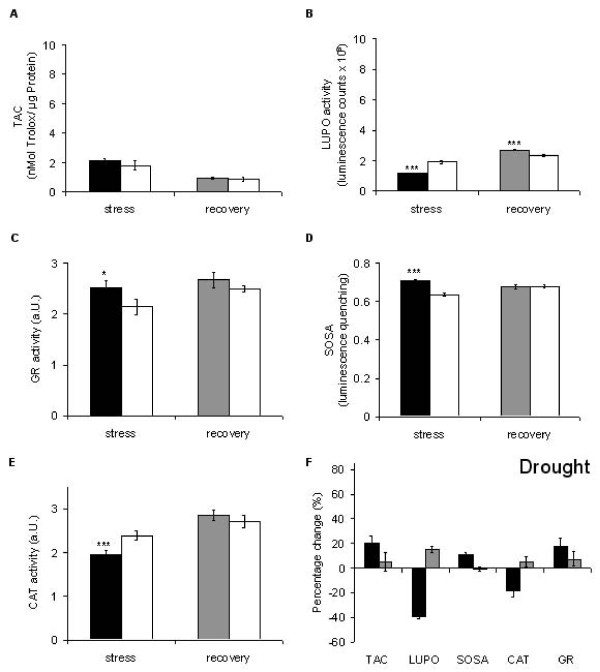
**Drought-induced alterations in the antioxidative system of *Lepidium sativum***. Drought was induced by withdrawal of nutrient medium for 24 h and antioxidative parameters were measured (black bars) in comparison to untreated plants (white bars). The grey bars represent the recovery status 24 h after restoring the nutrient medium. Data presentation as in Figure 5.

Gogorcena *et al*. [78] demonstrated that in pea (*Pisum sativum*) nodules drought causes a decrease in all relevant antioxidative enzymes and markers. They conclude that the decline of antioxidative capacity is due to an exhausted NAD(P)H pool. In contrast, Moran *et al*. [79] and Zhang and Kirkham [8] both reported an up-regulation of peroxidases and down-regulation of CAT in response to drought. The former is in line, the latter opposite to our findings (Figure 8B, E). However, the results are hardly comparable since their experiments [79] were performed on a different time scale (days) compared with those presented here (hours). As with heat treatment (Figure 6F) drought induced different effects on the various antioxidative parameters (Figure 8F) so that the area of the corresponding polygon remains almost unchanged (Figure 4C). The increase in SOSA (Figure 8D and Figure 4C) is also seen with heat and salt treatment (Figures 6D, 4B and Figures 7D, 4D). All three stressors produce an increase in cellular ionic strength that may increase a demand for superoxide scavengers. The reduction in LUPO (Figures 8B, F and Figure 4C) is in contrast to [79] and [8], suggesting either a loss of function at high ionic strength or a down-regulation due to a decreased demand of peroxidase activity in *Lepidium*. To decide this, we performed the LUPO assay in buffers of different ionic strengths (5 to 500 mM NaCl) and found no loss of function at high salt concentrations (data not shown). Therefore, a demand-driven down-regulation is more likely. This conclusion is also valid for salt stress (Figures 4D, 7B). The slight expansion of the polygon after recovery (Figure 4C, grey polygon) suggests a formation or mobilisation of over-all antioxidative power. This may constitute a memory for future challenges.

### Conservation of resources rules adaptation and leads to a shift of functions

The current understanding of metabolic adaptation in response to abiotic challenges assumes genetic induction and the formation and mobilisation of antioxidative power. From the work presented here, another facet of this response emerges, namely a shift of function, i.e. the down-regulation of a selected function in favour of another.

If abiotic stress challenge only led to the formation of antioxidative power, then this would appear as an increase in area when plotting the changes in any set of screened parameters as a polygon. This is true only for cold stress treatment (Figure 4A). For all other abiotic stimuli no remarkable increases in area are seen (Figures 4B–D). Here, any expanding corner representing an up-regulated parameter is compensated by a contraction elsewhere in the polygon representing a down-regulation. This is particularly obvious when comparing LUPO and SOSA. These two parameters run anti-parallel under all investigated stress conditions, i.e. SOSA is increased while LUPO is down-regulated and *vice versa*. This is reasonable, since SODs form H_2_O_2 _and peroxidases may also produce superoxide [39].

This also suggests that the plant is limited in mobilising resources under stress and mainly responds to abiotic stress by shifting antioxidative functions, i.e. up-regulation of necessary scavengers and a down-regulation of dispensable functions. Consequently, the fingerprint may yield information as to what kind of ROS is mainly involved in each type of abiotic stress. Thus, if there is no increase in CAT, then there is probably no demand for additional H_2_O_2 _scavengers. Conversely, if there is a shift towards SOSA then superoxide anions may be the major ROS.

### Redundancies in the fingerprint

Here, we show that each kind of abiotic stress gives rise to a specific antioxidative defence (Figure 4). However, redundancies within the group of screened parameters reduce the informative value. Therefore it is critical to identify these potential redundancies.

Among the five parameters presented here (i.e. TAC, LUPO, SOSA, CAT, GR) redundancies appear to be present: TAC and GR always run in parallel. This coincidence suggests that the majority of low molecular weight antioxidants (i.e. TAC) in the plant is represented by GSH. Further, if an interdependency of LUPO and SOSA can be consolidated then their anti-parallel behaviour could also be regarded as redundant.

On the one hand low redundancy is helpful within a multi-parametric set of assays to increase the informative value. On the other hand, however, some redundancy can be used as an internal cross-check to identify errors and to minimise misinterpretation.

### The antioxidative system depends on circadian rhythm

Differences in controls (i.e. plants not challenged with abiotic stress, shown by white bars in Figure 5, 6, 7, 8) can be attributed to developmental and circadian variations. For instance, controls running in parallel with the heat stress experiments were harvested at midday and in the evening (6 h interval). They show considerable differences in all screening parameters. In particular, LUPO is up-regulated at midday, when maximum photosynthesis (ROS production) is reached, and down-regulated in the evening. Differences are less obvious in the controls of the salt and drought stress experiments (Figures 7, 8 white bars). Here, all samples were harvested at the same time of the day. In contrast, the controls of the cold stress experiments have been harvested in 12 h intervals (evening and morning). Here, GR activity in the 'morning sample' (Figure 5C, last column) is slightly higher than in the 'evening sample' (Figure 5C, second column). This could be an indication for circadian variations of GR activity, as has been discussed previously [80].

### Fingerprinting complexity

Antioxidative compounds can be very different among plant species [81]. Hence, it is more meaningful to monitor a global parameter such as TAC before going into a detailed analysis. Nevertheless, many other plant metabolites with specific antioxidative properties can also be studied separately. However, the analysis of each component requires specific processing of plant material and analytical methods. A gain in information can justify expenses and effort, but, as mentioned above, an increase in specificity would also generate more redundant data.

### Flexibility and memory

Apart from salt stress (Figure 4D, discussed above) the areas of all grey polygons in Figure 4, representing the antioxidative status after recovery, are close to the red pentagons (i.e. zero alterations). This shows that the antioxidative system is very flexible and that any changes are quickly adjusted to normal when stress factors are removed. Nevertheless, heat (Figure 4B) and drought (Figure 4C) show a slightly increased polygon area even after recovery. This could be interpreted as a gain in tolerance and is in line with findings that abiotic stress stimuli leave an imprint on plant metabolism [76,82].

### Tolerance and robustness

Robustness of a plant is defined as the ability not only to withstand adverse conditions but also to propagate, and to produce a yield of sufficient quality in non-optimal environments. Tolerance to abiotic stress and robustness have often been directly linked to the efficiency of the antioxidative system ([83] and references cited therein; [84]). A more robust plant is therefore able to activate its antioxidative systems faster, or to shift antioxidative functions more effectively.

### Future prospects – breeding and screening

There have been many attempts to breed robust, stress tolerant crop plants, even by transgenic approaches (; [85-87]). The success of this endeavour has to be verified by long lasting and costly growth-yield experiments. However, if a certain pattern or fingerprint of the antioxidative system, as depicted here (Figure 4), could be shown to strictly correlate with robustness, then screening would become easier. In plant breeding projects high throughput screening is needed to distinguish stress tolerant from sensitive breeds at an early stage of growth [88]. Thus, metabolic 'summary parameters' may be implemented as quantitative traits when screening for robustness among different ecotypes, novel transgenics or breeds of a plant species.

Directed genetic interference with the antioxidative system sometimes leads to improvement of stress resistance [89]. However, unexpected results are often obtained [90-93], requiring detailed analysis of what has changed in the overall metabolism. In such cases, a fingerprinting approach could also help to find explanations.

## Conclusion

Here, we provide a set of 'summary parameter' assays which can be run in parallel with a minimum of effort necessary for plant tissue preparation and processing and at low cost. TAC delivers valuable data about the presence of low molecular weight antioxidants in general. LUPO displays all luminol converting peroxidase activities in the sample. SOSA quantifies high molecular weight superoxide anion scavengers.

Together with common CAT and GR assays, we obtained specific fingerprints from *Lepidium sativum *for each type of abiotic stress situation. The highly flexible antioxidative system of plants is mainly based on functional shifts rather than costly formation of new antioxidative resources.

The approach reported here may help to detect and fingerprint plant robustness. Generic parameters like TAC, SOSA and LUPO have the potential to be developed as tools for high- throughput screening the robustness of novel mutants, transgenics, or breeds.

## Methods

### Chemicals

Bradford Assay Reagent (BioRad #500-0069)

Calcium chloride (CaCl_2 _6 H_2_O; Riedel deHaen #12074; MW = 220 g/mol)

Coelenterazine (NanoLight Technologies #303; MW = 423 g/Mol)

Di-potassium hydrogen phosphate (Roth #T875; MW = 174 g/Mol)

EGTA (Sigma #E4378; MW = 340 g/Mol)

Ethanol abs. (Roth # 9065)

Glutathione – oxidised (GSSG; Roth #6378; MW = 612 g/Mol)

Horseradish peroxidase (HRP; Sigma #P6140, ca. 2 kU/ml)

Hydrochloric acid (HCl; Roth #4625; 1 M, i.e. 34% 1:10 diluted in H_2_O)

Hydrogen peroxide solution (H_2_O_2_; Merck # 1.08597; 30% = 8.8 M; MW = 34 g/Mol)

Hypoxanthine (Fluka #56700; MW = 136 g/Mol)

Iodophenol (Fluka #58020, MW = 220 g/Mol)

Luminol (Fluka #09253; MW = 177 g/Mol)

Murashige and Skoog (M&S) medium (Duchefa, #M0222)

NADPH tetra-sodium salt (Roth #AE14; MW = 833 g/Mol)

Potassium di-hydrogen phosphate (Merck #1.04873; MW = 136 g/Mol)

Potassium hydroxide (KOH; Roth #6751; MW = 56 g/Mol; 5 M in H_2_O)

Sodium chloride (NaCl; Fluka #71378; MW = 58 g/Mol)

TRIS ultra pure; (ICN Biomedicals #77861; MW = 121 g/Mol)

Triton^® ^X-100 (Sigma #X100; MW = 647, Liquid ρ = 10.7 kg/L ≅ 1.65 M)

Trolox (Aldrich #23,881; MW = 250 g/Mol)

Xanthine oxidase (from bovine milk; Sigma #X4500)

### Plant growth

Seeds from *Lepidium sativum *were obtained from Sperli (#42.953, Germany, ). For surface sterilization 4 gram of seeds were vortexed 2 min. in 80% ethanol and dried on filter paper.

For cold and heat treatment seeds were sown on 2 layers of synthetic capillary matting (medical fleece "Rolta^®^-soft" # 932048, Hartmann, Germany, ) soaked with 0.5 × Murashige and Skoog medium (Duchefa #M0222). Seedlings were grown in a mini propagator (26 × 11 × 7 cm, Windhager^®^; Austria; ) at 21°C with a 16 h photoperiod (50 μE, white fluorescent lamps Osram L36 W/77) for five days before treatment with abiotic stress.

For salt and drought treatment plants were hydroponically grown on 0.5 × M&S medium in sterile Growtek™ culture vessels (#BEL1768; BLD Science^®^, NC, USA; ) as above. Hydroponical growth enables easy removal and exchange of nutrient medium.

### Plant stress treatment

Cold and heat stress were applied by placing the mini-propagator for 12 h at 0°C or 6 h at 42°C, respectively, in the dark. Recovery times were allowed for 12 h and 6 h, respectively, in the growth room. Whole plants were harvested and processed.

Salt stress was induced by exchange of the nutrient medium with 150 mM NaCl in 0.5 × MS medium. Salinity was applied for 24 h. For recovery, the salt solution was removed. Roots were rinsed with tap water before plants were allowed to recover for 24 h in 0.5 × MS medium and under conditions as before.

For drought stress, the nutrient medium was completely withdrawn for 24 h. This period is sufficient to produce an appreciable limp appearance and yet short enough to maintain ability for full recovery. For recovery (24 h) 0.5 × MS medium was replaced. From plants grown in Growtek™ vessels only the green parts were harvested and processed.

### Processing plant material

Plant material was harvested after stress treatment and after recovery time. Untreated controls were run in parallel (experimental design see Fig. 4.1 in additional file 4). Portions of 4 grams fresh weight were snap-frozen in liquid nitrogen and stored at -80°C until processed.

During further processing (see Fig. 4.2 in additional file 4) contact of samples with ambient oxygen was minimised. Frozen plant material was ground in liquid nitrogen with a mortar and pestle. Ten volumes ice-cold degassed buffer (100 mM TRIS/HCl pH 8.6 + 2 mM CaCl_2 _+ 1 mM Triton^® ^X-100) was added to the powder (ml/g), vortexed for 2 min, and filtered through a fluted paper filter in a 0°C cabinet under N_2_-atmosphere. This crude extract was aliquotted for membrane filtration (TAC) and for dialysis (LUPO, SOSA, enzyme assays).

For analysis of low molecular weight total antioxidative capacity (TAC) the crude extract was passed through a membrane filter (MWCO = 10 kDa, VivaSpin 20, VivaScience, Germany, ) by centrifugation at 0°C.

Since low molecular weight antioxidants and phenolics can interfere with enzymatic assays of LUPO, SOSA, CAT, and GR [94], these compounds were removed by dialysing the crude extract in dialysis membrane tubing (MWCO = 10 kDa; Roth #E668.1) for 30 min per 100 μl dialysate against twohundred-fold volume of ice-cold assay buffer.

Assay results have to be displayed with reference to an invariable parameter. Therefore, in many previous studies plant fresh weight or dry weight was used. However, dry weight is not invariable with salt treatment due to considerable ion uptake and fresh weight may vary with drought treatment due to loss of water. Consequently, we employed the total protein content as a reference parameter, knowing that this may also vary within certain limits. Thus, after dialysis the protein concentration was determined by the Bradford assay [95]. Before assaying, all samples were supplemented with buffer in order to equalise differences in protein concentrations.

### Assay procedures

Luminescence assays were performed with a simple chemiluminometer (PMT 9829A, Electron Tubes Ltd. Ruislip, UK) equipped with a light tight sample housing to hold vials in front of the detector [96].

### TAC: HRP-based total low-molecular-weight antioxidative capacity assay

The assay buffer was 1 mM CaCl_2 _+ 100 mM TRIS/HCl pH 8.6. Iodophenol was dissolved in ethanol to give a 100 mM colourless stock solution. Luminol dissolved in 5 M KOH gave a 1 M stock solution. The assay mixture (sufficient for ca. 2000 samples) was prepared by adding 20 μl ethanolic iodophenol stock, 500 μl luminol stock, 100 μl HRP suspension, and 660 μl Triton^® ^X-100 to 1 L of assay buffer. This mixture can be used diluted to increase assay sensitivity and thereby meet samples with lower TAC (Fig. 1.6 in additional file 1). It is stable for several weeks, when stored at 4°C (Fig. 1.7 in additional file 1). H_2_O_2 _(1.1 mM) solution was prepared by diluting H_2_O_2_-stock (30%) 1:8000 in 100 ml of assay buffer. The light emitting reaction was started by mixing 0.5 ml H_2_O_2 _with 0.5 ml of assay mixture. After establishment of a constant signal, the luminescence was quenched by injecting the sample (0.5 ml). Light recording was continued until luminescence recovery (details in additional file 1).

### LUPO: Luminol-based peroxidase assay

An assay buffer of 100 mM TRIS/HCl pH 8.6 + 2 mM CaCl_2 _+ 1 mM Triton^® ^X-100 was used. Luminol (1 mM) solution was prepared by diluting 1 M alkaline luminol stock solution in assay buffer. A 17.6 mM H_2_O_2 _solution was prepared by diluting H_2_O_2 _(30%) 1:500 in assay buffer. Dialysed samples were diluted 1:100 in assay buffer. 0.5 ml of diluted sample was mixed with 0.5 ml luminol solution and background luminescence was recorded. The light reaction was started by adding 0.5 ml 17.6 mM H_2_O_2 _solution. Counts per second (cps) were recorded for several minutes and light output integrated (details in additional file 2).

### SOSA: CTZ-based superoxide anion scavenging activity assay

An assay buffer containing 100 mM potassium phosphate pH 7.4 + 0.1 mM EDTA + 6 mM Triton^® ^X-100 was used. CTZ (50 μM) solution was prepared by diluting a 5 mM methanolic stock solution in thoroughly de-gassed potassium phosphate buffer (100 mM pH 7.4). CTZ solution was aliquotted to 0.25 ml portions in luminometer vials under an oxygen free atmosphere and capped. Hypoxanthine (1 M) stock solution was prepared in 5 M KOH. HX (1 mM) was prepared by dilution this alkaline stock 1:1000 in assay buffer. Xanthine oxidase mix was prepared by diluting XOD suspension 1:3000 in assay buffer. Dialysed samples were diluted 1:8 in assay buffer. Dark-background from uncapped CTZ aliquots was recorded before injecting 0.25 ml HX-solution. Background luminescence was recorded for a while and then 0.5 ml XOD-mix was injected to initiate the superoxide-CTZ reaction. 0.5 ml of diluted dialysed sample was injected to quench luminescence and light recording was continued for several minutes. Before assaying for SOSA the dialysate was checked for TAC to ensure that no low molecular weight antioxidants adulterate the assay.

### Catalase assay

The assay buffer consisted of 100 mM potassium phosphate pH 7.4 + 8.8 mM H_2_O_2_. The CAT-assay was based on the UV-absorption of H_2_O_2 _[97]. The consumption of H_2_O_2 _after injecting the CAT-containing sample was recorded at λ_ABS _= 240 nm in a spectrophotometer (Ultrospec 2100pro, GE-Healthcare Europe, München, Germany). The linear decay in H_2_O_2 _was calculated (Figures 5E, 6E, 7E, 8E).

### Glutathione reductase assay

An assay buffer containing 100 mM potassium phosphate pH 7.4 + 0.1 mM NADPH + 1 mM GSSG was used. The GR-assay is based on the absorption of light by NADPH, the co-substrate of the GR [98]. The fall in absorption of λ_ABS _= 340 nm was recorded with a spectrophotometer (as above) after injecting the GR containing sample. The linear decay of NADPH signal was calculated (Figures 5C, 6C, 7C, 8C).

## Abbreviations

APX: ascorbate peroxidase; Avrg: average; CAT: catalase; cps: counts per second; FW: fresh weight; GPX: glutathione peroxidase; GR: glutathione reductase; GSH: glutathione; HRP: horseradish peroxidase; LUPO: luminol converting peroxidase; MWCO: molecular weight cut-off; PO: peroxidase; ROS: reactive oxygen species; SOD: superoxide dismutase; SOSA: superoxide scavenging activity; StDv: standard deviation; TAC: total antioxidative capacity.

## Competing interests

The authors declare that they have no competing interests.

## Authors' contributions

LS carried out the experiments reported in the main manuscript, performed data processing and statistical analysis, and participated in amending the draft. CP conceived of the study, carried out the experiments shown in the additional files, and wrote the manuscript. Both authors approved the final version.

## Supplementary Material

Additional file 1 **The TAC Assay.** The data provide information about optimal assay conditions for maximal light output. Further information is given for calibration and tuning the assay sensitivity. Fig. 1.1 The light emitting luminol reaction. Fig. 1.2 Enhanced *versus *not enhanced HRP-catalysed luminol reaction. Fig. 1.3 The pH-optimum of the HRP-catalysed luminol reaction. Fig. 1.4 The peroxide inactivation or 'suicide reaction'. Eq. 1 Definition Quenching. Fig. 1.5 Calibration of luminescence recovery times. Fig. 1.6 Tuning the TAC assay sensitivity. Fig. 1.7 Stability of the TAC assay mix Click here for file

Additional file 2 **The LUPO Assay. **The data provide information about optimal assay conditions for maximal light output. Further information is provided about H_2_O_2_-sensitivity, calibration in terms of a purified peroxidase, and the heat sensitivity of *Lepidium *LUPOs. Fig. 2.1 The luminol converting peroxidase (LUPO) cycle. Fig. 2.2 Peroxidases from *Lepidium sativum *are not inactivated by H_2_O_2_. Fig. 2.3 The luminol reaction catalysed by purified lignin peroxidase. Fig. 2.4 Total light yield of the non-enhanced luminol reaction. Fig. 2.5 LUPOs from *Lepidium *are heat-sensitive. Click here for file

Additional file 3 **The SOSA Assay.** The data provide information how to calibrate the SOSA assay in terms of a purified superoxide dismutase. Further information is given about different coelenterazine analogues, their performance as superoxide indicators, and their optimal concentration in the SOSA assay. Fig. 3.1 Superoxide generation and coelenterazine-mediated light emission. Tab. 1 Coelenterazine analogues tested for the SOSA assay. Fig. 3.2 The luminescence quenching correlates with SOSA. Fig. 3.3 The CTZ analogue is crucial for the SOSA assay performance. Fig. 3.4 CTZ concentration determines the duration of light output. Click here for file

Additional file 4 **The experimental design.** The figures summarize the chronology of plant growth, treatment, and harvest and give a scheme how to process the biological material. Fig. 4.1 Experimental design. Fig. 4.2 Flow chart for processing plant material Click here for file
