# Supplementary material for: Fingerprinting antioxidative activities in plants
Source: Plant Methods. 2009 Jan 26;5:2. doi: 10.1186/1746-4811-5-2 (PMC2656482; doi:10.1186/1746-4811-5-2)
Supplement: Additional file 1 — The TAC Assay. The data provide information about optimal assay conditions for maximal light output. Further information is given for calibration and tuning the assay sensitivity. Fig. 1.1 The light emitting luminol reaction. Fig. 1.2 Enhanced versus not enhanced HRP-catalysed luminol reaction. Fig. 1.3 The pH-optimum of the HRP-catalysed luminol reaction. Fig. 1.4 The peroxide inactivation or 'suicide reaction'. Eq. 1 Definition Quenching. Fig. 1.5 Calibration of luminescence recovery times. Fig. 1.6 Tuning the TAC assay sensitivity. Fig. 1.7 Stability of the TAC assay mix [file 1746-4811-5-2-S1.pdf]

The TAC Assay

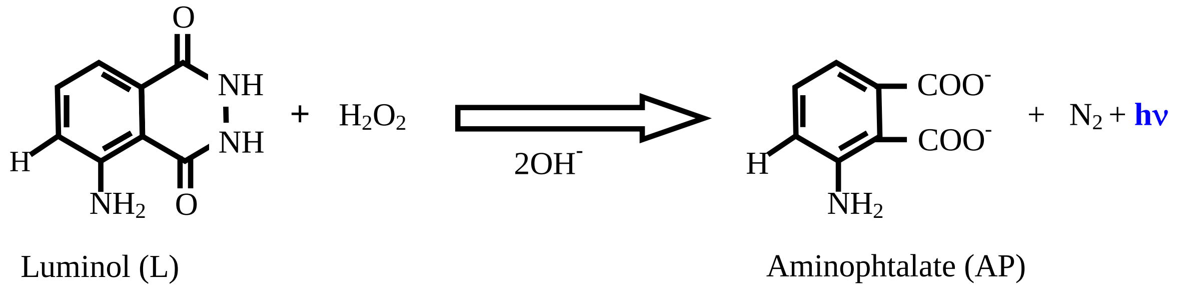

Figure 1.1 The light emitting luminol reaction
Oxidation of luminol under alkaline conditions produces blue chemiluminescence.

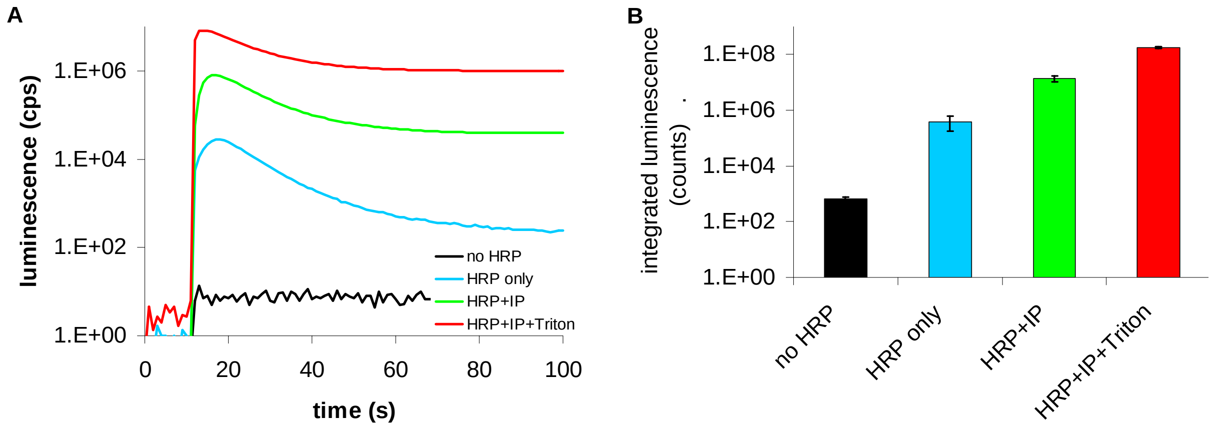

Figure 1.2 Enhanced *versus* not enhanced HRP-catalysed luminol reaction. The light yield from HRP-catalysed reactions depends on the presence of enhancers (here: 3-iodophenol = IP) and a surfactant (here: Triton-X-100). **A:** Light emission from different luminol reaction mixtures (0.5 ml) was triggered at t = 12 s by injecting 0.5 ml of 1.1 mM H_2O_2 and recorded for 100 s. **B:** The light yield (integrated for 100 s) is displayed for four different luminol reaction mixtures. Averages of n = 3. Error bars represent standard deviation.

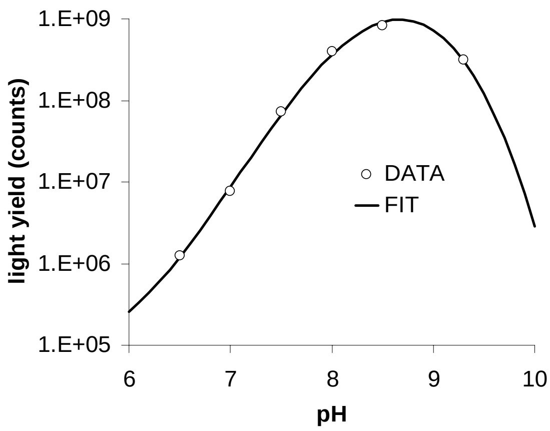

Figure 1.3:
The pH-optimum of the HRP-catalysed luminol reaction is around pH 8.7.
HRP mix and H_2O_2 were adjusted to the desired pH. Reactions were started by mixing 0.5 ml HRP mix with an equal amount of 1.1 mM H_2O_2 of same pH. The integrated light output is plotted *vs.* pH. Averages of n = 3. StDv is within symbol size.

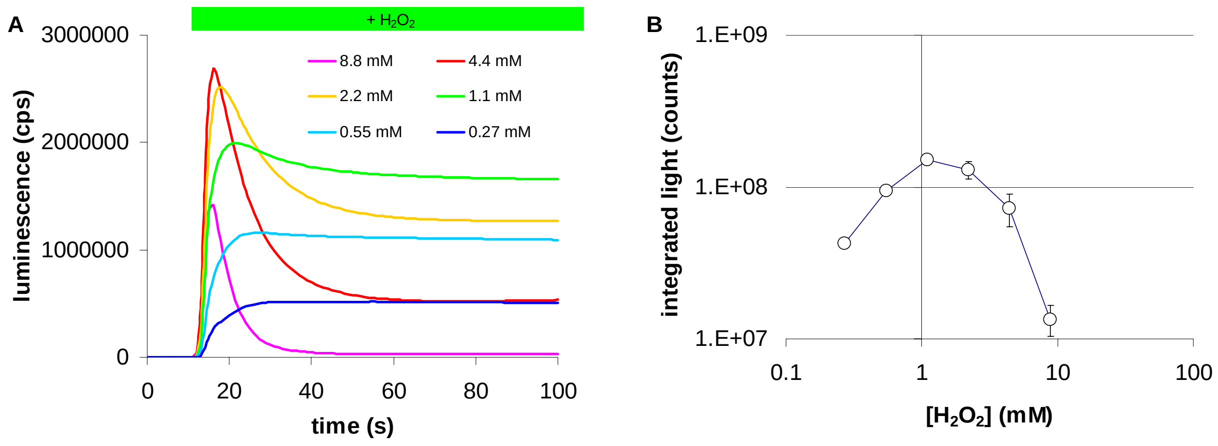

Figure 1.4 Dependence of light yield from enhanced HRP-catalysed luminol reaction on H_2O_2 concentration: The peroxide inactivation or 'suicide reaction'. A: Light output of enhanced HRP-luminol reactions during the first 90 sec. The reactions were triggered by mixing 0.5 ml of HRP mix with 0.5 ml H_2O_2 (concentrations given in the legend) in Tris-buffer at t = 12 s. The curves show that the ratio of HRP to H_2O_2 needs to be well balanced for maximum light yield. Insufficient H_2O_2 gives a stable long lasting glow, though at a low level. Too much H_2O_2 gives a short light pulse before the HRP is inactivated by suicide inhibition. **B:** Plot of integrated light yield (= sum of counts during the first 90 sec of reactions shown in A) over the concentration of added H_2O_2 in log-log scale. The optimal [HRP]:[H_2O_2] ratio for maximal light yield is [200 U L^{-1}]:[1 mMol L^{-1}]. Traces given in A are averages of n = 4 with StDv of less than 15 % of peak value. Averages in B are calculated from data shown in A. Error bars in B give standard deviation if not within symbol size.

Definition Quenching:

$$Q := \frac{\log(MAX) - \log(MIN)}{\log(MAX)} \qquad \textbf{Eq. 1}$$

MAX := maximum light output after addition of H_2O_2;
MIN := minimum light output after sample injection.

Tuning the TAC assay sensitivity

The sensitivity of the TAC assay can be increased by lowering HRP and H_2O_2 concentration in the assay mixture and the H_2O_2-starter solution, respectively. The ratio of [HRP]: [H_2O_2] should thus be kept constant to avoid peroxide inhibition of HRP (**Fig. 1.4**). At the same time the photodetector sensitivity (e.g. PMT high voltage) must be adjusted to keep the signal response at reasonable dynamic range (**Fig. 1.6**)

Tuning the assay sensitivity is important in several respects:

1. The desired range of sensitivity must cover the range of TACs in the set of samples to be screened. In particular samples with low TAC do not produce sufficient signal quenching and reasonable recovery times. This can be overcome by increasing the assay sensitivity. This way statistical identity of biological replicates or significant differences between samples of different treatments can be worked out.

2. The time for screening a set of samples must be kept short. To achieve this, the time of signal recovery of the samples should ideally be between 60 and 600 sec.

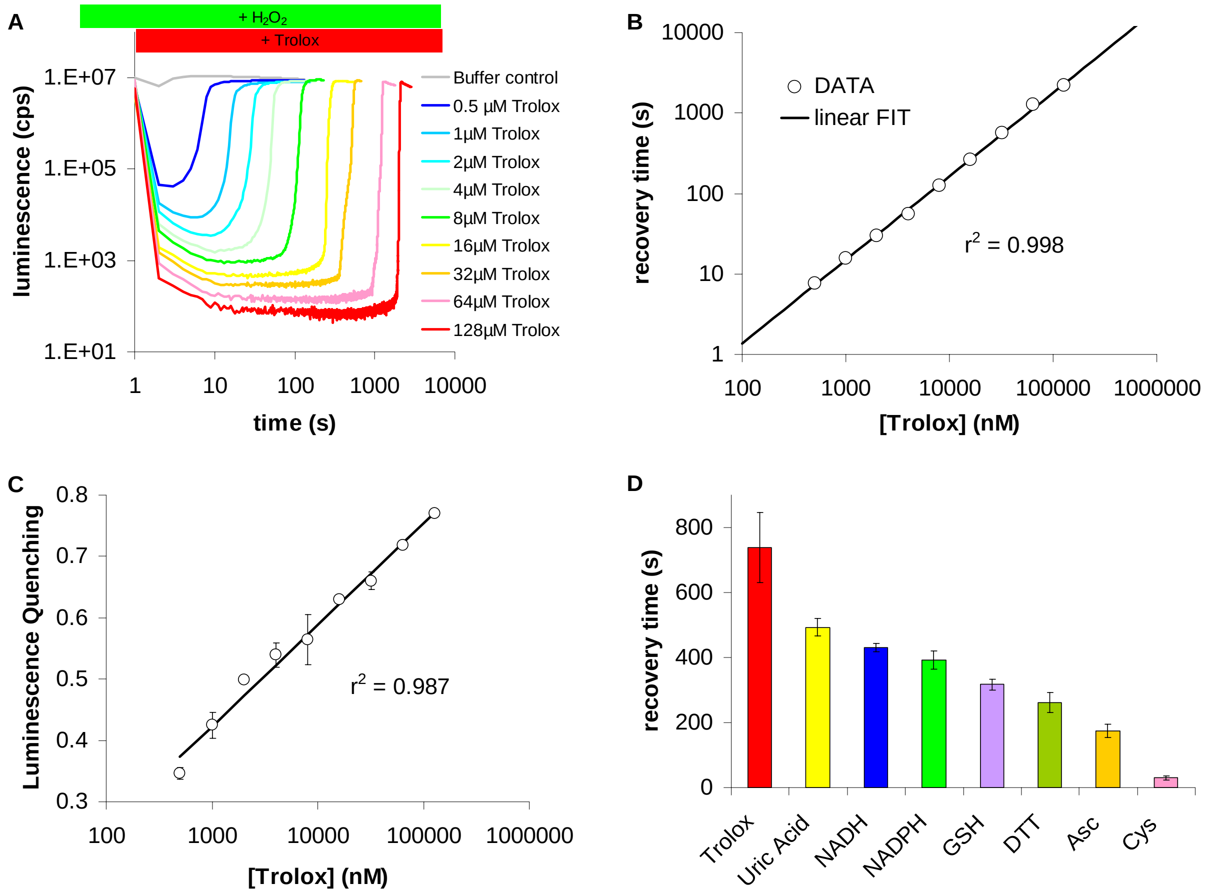

Figure 1.5 Calibration of luminescence recovery times and luminescence quenching in terms of equivalent trolox concentration. **A:** Both the time of signal recovery and signal quenching depend on the amount of antioxidant. **B:** The recovery time is linearly correlated with the logarithm of antioxidant concentration. Here averages of three technical replicates are given. The standard deviation (StDv) is within the symbol size. **C:** The luminescence quenching (as defined by **Eq. 1**) is also correlated with the logarithm of antioxidant concentration as well. Averages of three technical replicates are given; error bars represent StDv. **D:** Comparison of antioxidative capacities of different compounds. Given are the recovery times at 10 µM concentrations. Means of n = 5 replicates; error bars represent StDv

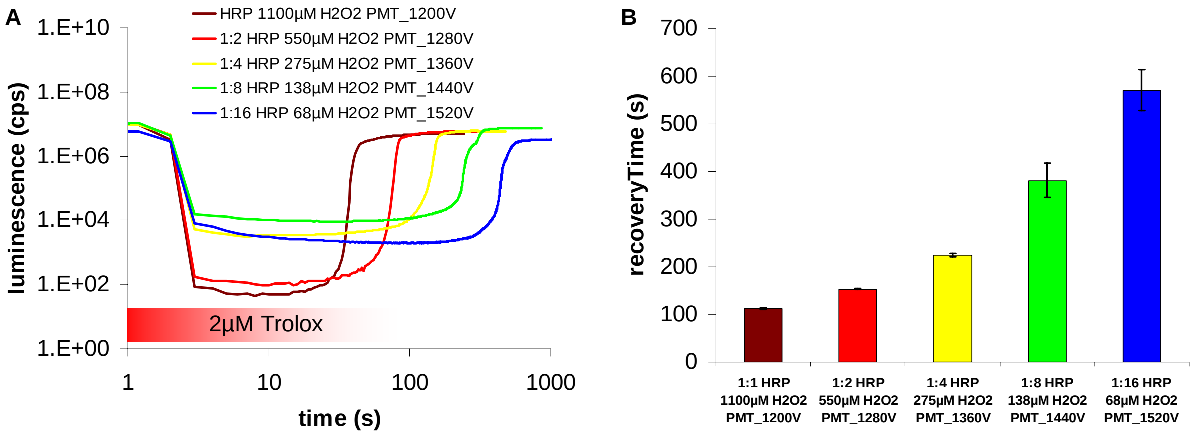

Figure 1.6 Tuning the TAC assay sensitivity. **A:** Both, HRP and [H_2O_2] were diluted in steps of 1:2 and PMT voltage increased in steps of 80V in parallel with each dilution to maintain signal output. 2 µM Trolox was injected at t = 1 s. The dilution of the assay causes the Trolox to live longer in the assay but requires increased sensitivity of the light detector. (Note: log-log scale!) **B:** Recovery times are plotted for each assay sensitivity (averages of n = 3; error bars represent standard deviation)

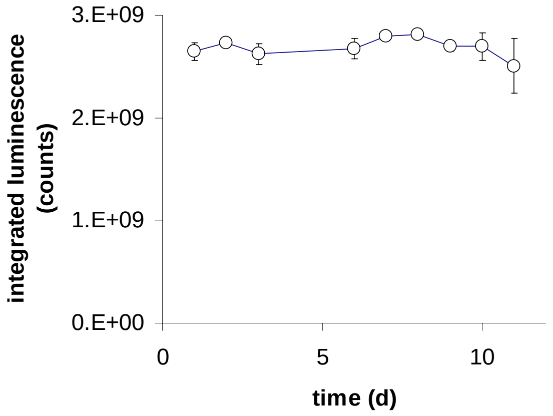

Figure 1.7 Stability of the TAC assay mix after storage for several days. The light yield (i.e. luminescence counts integrated over 5 min after starting the reaction with H_2O_2) of the enhanced luminol reaction is plotted. The mix is stable for several weeks when kept at 4°C. Even after two months there is still sufficient activity for TAC assays. Data represent averages of n = 3. Error bars represent StDv or are within symbol size.
