# Supplementary material for: Fingerprinting antioxidative activities in plants
Source: Plant Methods. 2009 Jan 26;5:2. doi: 10.1186/1746-4811-5-2 (PMC2656482; doi:10.1186/1746-4811-5-2)
Supplement: Additional file 2 — The LUPO Assay. The data provide information about optimal assay conditions for maximal light output. Further information is provided about H2O2-sensitivity, calibration in terms of a purified peroxidase, and the heat sensitivity of Lepidium LUPOs. Fig. 2.1 The luminol converting peroxidase (LUPO) cycle. Fig. 2.2 Peroxidases from Lepidium sativum are not inactivated by H2O2. Fig. 2.3 The luminol reaction catalysed by purified lignin peroxidase. Fig. 2.4 Total light yield of the non-enhanced luminol reaction. Fig. 2.5 LUPOs from Lepidium are heat-sensitive. [file 1746-4811-5-2-S2.pdf]

The LUPO Assay

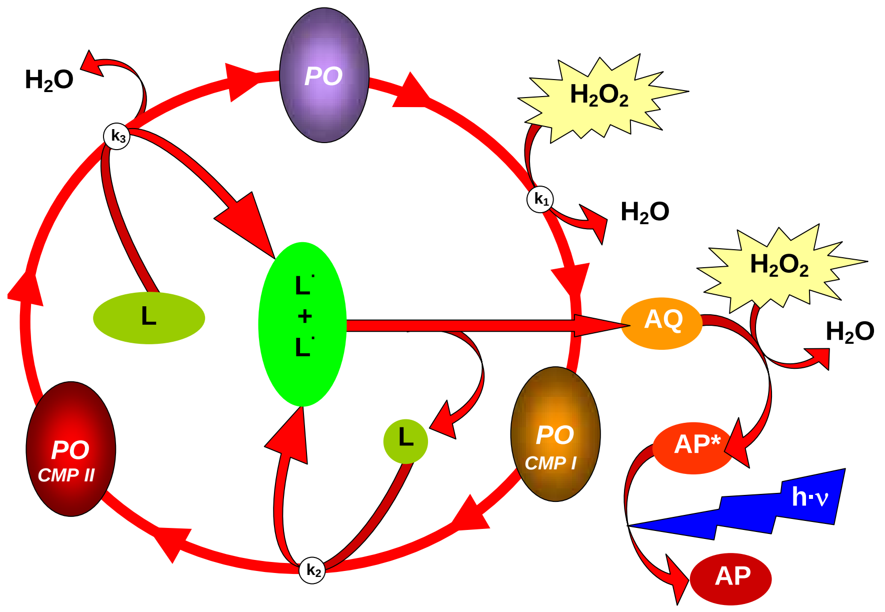

Figure 2.1 The luminol converting peroxidase (LUPO) cycle.
Luminol (L) is accepted as substrate by many plant peroxidases (PO). Hence, the light generating process can be used for quantification of peroxidase activity. As with the HRP cycle (**Fig. 1** in main manuscript) a di-aza-quinone (AQ) is formed as intermediate. This in turn is oxidised by hydrogen peroxide (H_2O_2) to form an excited state of aminophtalate (AP*). The final step is the emission of blue (420 nm) light (***h·ν***) when the excited AP* returns to its ground state. However, in contrast to the HRP cycle (**Fig. 1**), there is no inactivation of the light emitting reaction at higher H_2O_2 concentrations (**Fig. 2.2**).

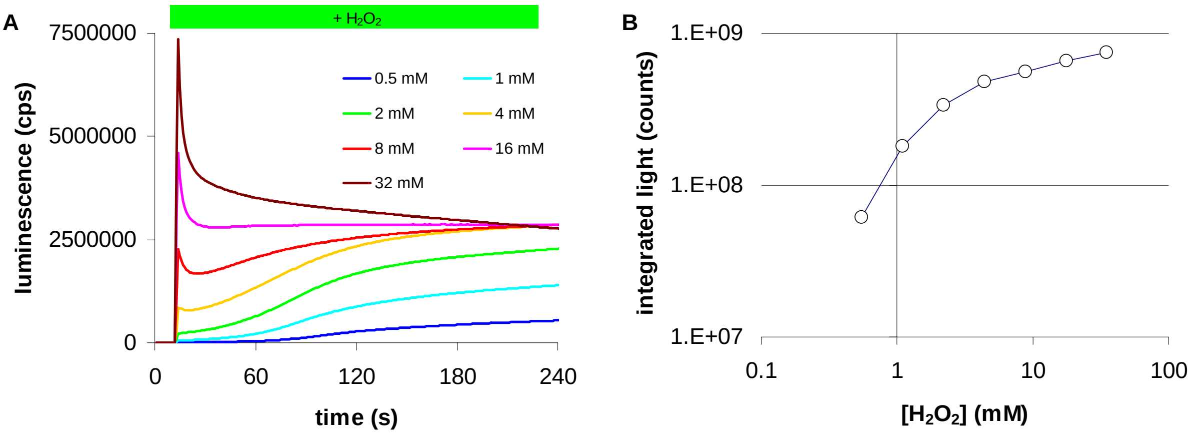

Figure 2.2 Luminol converting peroxidases from *Lepidium sativum* are not inactivated by H_2O_2
A: Light output of reactions during the first 4 min. The reactions were triggered by adding 0.5 ml H_2O_2 (concentrations given in the legend) to 1 ml of peroxidases/luminol assay mix at t = 12 s. The curves show that light yield increases with increasing H_2O_2 concentration. *Lepidium* peroxidases appear to withstand even high H_2O_2 concentrations when compared with HRP (**Fig. 1.2**). **B:** Plot of integrated light yield (= sum of counts during the first 240 sec of reactions shown in A) against the concentration of added H_2O_2 in log-log-scale. Traces given in A are averages of n = 3 with StDv of less than 15% of peak value. Averages in B are calculated from data shown in A. Error bars are equal or below symbol size.

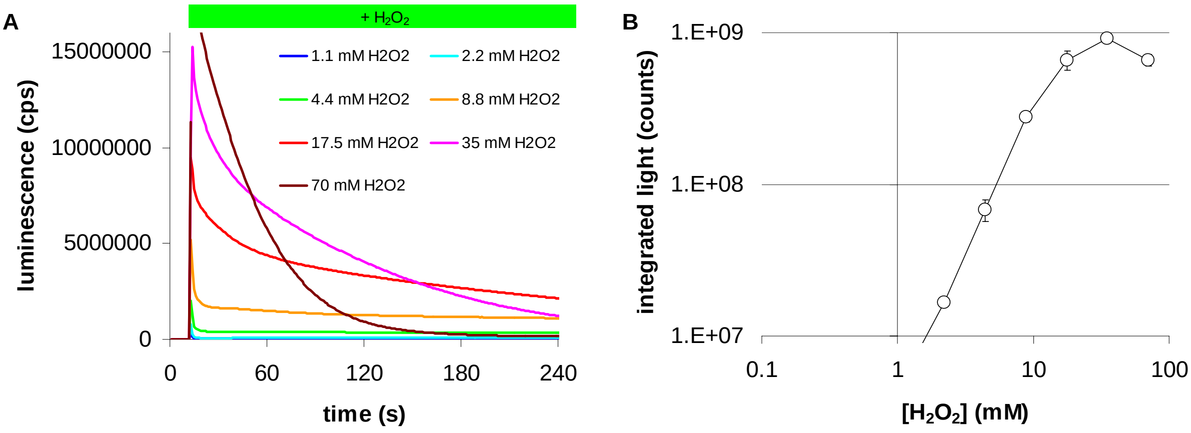

Figure 2.3 The luminol reaction catalysed by purified lignin peroxidase from *Phanerochaete chrysosporium* (Fluka #42603) **is inactivated only at very high H_2O_2 concentrations. A:** Light output of reactions during the first 4 min. The reactions were triggered by adding H_2O_2 (concentrations given in the legend). Lignin peroxidases appear to withstand H_2O_2 concentrations up to 35 mM when compared with HRP (see **Fig. 1.2**). **B:** Integrated light yield calculated from A plotted over the concentration of added H_2O_2 in log-log scale. Traces in A are averages of n = 3 with StDv of less than 15 % of peak value. Error in B bars are equal or below symbol size.

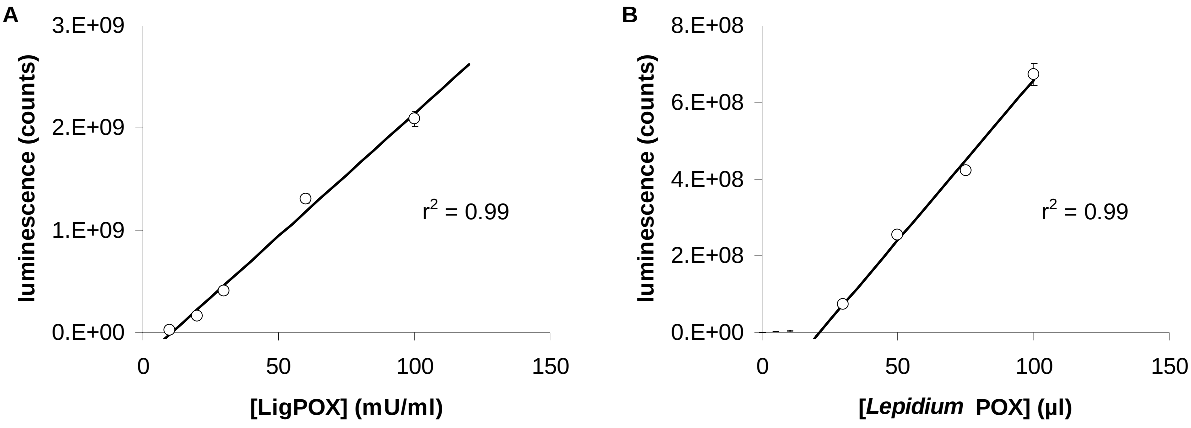

Figure 2.4 Total light yield of the non-enhanced luminol reaction is linearly correlated with the amount of the catalysing peroxidase. A: Integrated light output of the luminol reaction catalysed by a lignin-peroxidase (Fluka #42603) as a standard peroxidase is plotted over the peroxidase concentration. **B:** Integrated light output from different amounts of *Lepidium* LUPO. Averages of n = 5. Error bars - if not below symbol size - represent standard deviation.

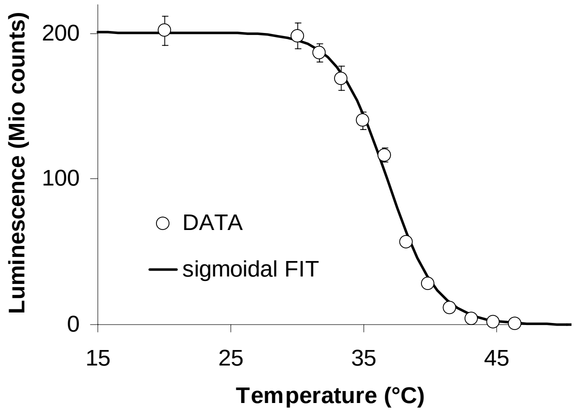

Figure 2.5 LUPOs from *Lepidium* are heat-sensitive. LUPOs from *Lepidium* were treated *in vitro* for 3 hours with the temperatures indicated in a PCR gradient cycler before light yield of the H_2O_2-luminol reaction was measured. The temperature of 50% inactivation is 36.7°C. Given are means of n = 5. Errorbars represent StDv or are below symbol size.
