# Supplementary material for: Fingerprinting antioxidative activities in plants
Source: Plant Methods. 2009 Jan 26;5:2. doi: 10.1186/1746-4811-5-2 (PMC2656482; doi:10.1186/1746-4811-5-2)
Supplement: Additional file 3 — The SOSA Assay. The data provide information how to calibrate the SOSA assay in terms of a purified superoxide dismutase. Further information is given about different coelenterazine analogues, their performance as superoxide indicators, and their optimal concentration in the SOSA assay. Fig. 3.1 Superoxide generation and coelenterazine-mediated light emission. Tab. 1 Coelenterazine analogues tested for the SOSA assay. Fig. 3.2 The luminescence quenching correlates with SOSA. Fig. 3.3 The CTZ analogue is crucial for the SOSA assay performance. Fig. 3.4 CTZ concentration determines the duration of light output. [file 1746-4811-5-2-S3.pdf]

The SOSA Assay

The suitability of lucigenin and luminol as probes for superoxide has been disputed [1-3]. Therefore, the superoxide scavenging assay presented here is based on coelenterazine (CTZ) luminescence. CTZ luminesces as soon it comes into contact with ambient oxygen. This is the background luminescence. Luminescence increases by a factor of up to 100 when superoxide is present (**Fig. 3** in main manuscript; **Fig. 3.1**). This increase is different with each CTZ variety, but it defines the dynamic response range of the SOSA assay (**Fig. 3.3**).

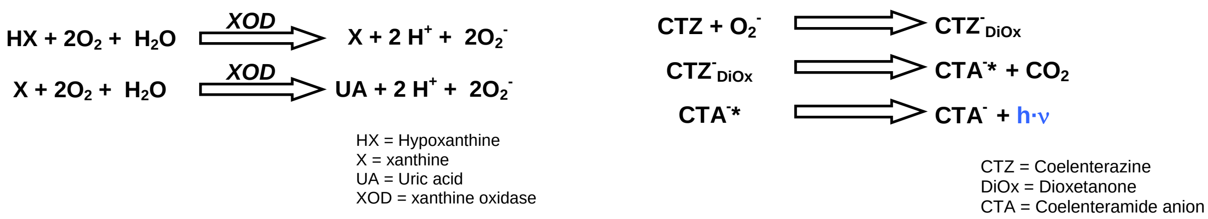

A B

Figure 3.1 Superoxide generation and coelenterazine-mediated light emission.
A: Superoxide anion production by Xanthine oxidase (XOD) converting hypoxanthine (HX) **B:** When coelenterazine (CTZ) is oxidised by superoxide to coelenteramide (CTA), blue light luminescence is produced. Adapted from [4].

Table 1 Coelenterazine analogues tested for the SOSA assay
The amount of XOD in the assay has no influence on the percentage of luminescence quenching by superoxide scavengers (Data not shown). The amount of luminescence quenching is linearly correlated with the log of scavengers added, regardless of whether the reaction is enzymatic (i.e. SOD) or non-enzymatic (**Figs. 3.2**).

CTZ analogue	other Name(s)	Source	Cat.-No.
CTZ	native coelenterazine; *Aequorea* luciferin	NanoLight Technologies, Pinetop, AZ, USA	# 303
dhCTZ	dehydroxy-coelenterazine, benzyl-coelenterazine, 2-deoxy-coelenterazine, *Renilla* luciferin; hCTZ	Biosynth AG, Staad, Switzerland	#C-7003
cpCTZ	cyclopentyl-coelenterazine	Invitrogen (Molecular Probes); Carlsbad, CA, USA	#C-14260
hcpCTZ	dehydroxy-cyclopentyl-coelenterazine		#C-14261
CTZ400a	DeepBlue-coelenterazine, Bis-deoxy-coelenterazine, di-dehydro-coelenterazine	Biotium Inc., Hayward, CA, USA	#10125

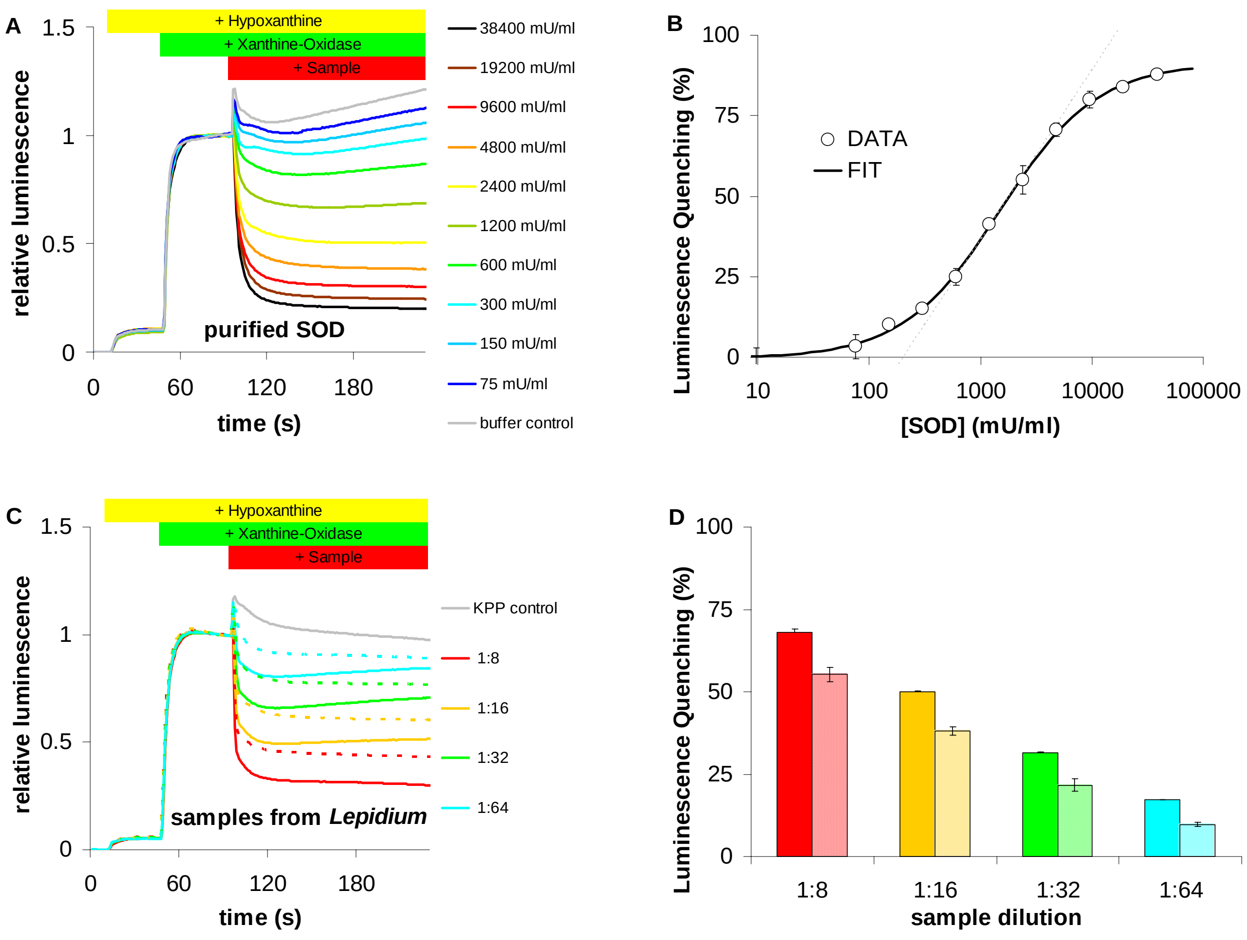

Figure 3.2 The luminescence quenching by SOSA correlates with the log of the amount of superoxide scavengers added.

A: Purified SOD from bovine liver (Sigma #S8409) in concentrations indicated in the legend is injected into the SOSA assay, mixed and luminescence quenching is recorded for 2 min. The steady state luminescence after starting the reaction with XOD (62 s < t < 72 s) was used to normalise the data. **B:** Luminescence quenching calculated from the data in A is plotted against the SOD concentration. The plotted parameters are linearly correlated when quenching is between 20 and 80 % ($r^2 = 0.996$). Data in B are averages of n = 3. Error bars represent StDv. **C:** Dialysed plant extracts from *Lepidium sativum* in various dilutions (specified in the legend) are added to the SOSA assay mix and luminescence quenching is recorded. The dotted lines represent corresponding dilutions of heat-inactivated samples. The steady state luminescence after starting the reaction with XOD (62 s < t < 72 s) was used to normalise the data. **D:** Luminescence quenching calculated from data in C is plotted. Light bars represent the corresponding heat-inactivated samples, i.e. the non-enzymatic superoxide scavengers. The differences between adjacent full and light bars represent the SOD activity in the sample. Data in D are means of n = 3. Error bars are StDv.

The CTZ analogue with the lowest superoxide-derived luminescence yield (i.e. CTZ 400a) also has the lowest background and thus provides the best dynamic range (**Fig. 3.3C**). Unfortunately, CTZ 400a is expensive compared to other available CTZ-analogues (for more details see [5, 6]). Native CTZ is the cheapest and gives both a good light yield and a good luminescence signal-to-background ratio, sufficient for all applications. CTZ is consumed in

presence of superoxide. Hence, the concentration of CTZ in the SOSA assay defines the duration of constant light output (**Fig. 3.4**). Thus, for a cost-effective procedure the concentration of CTZ used should be limited to the duration needed to assay a sample.

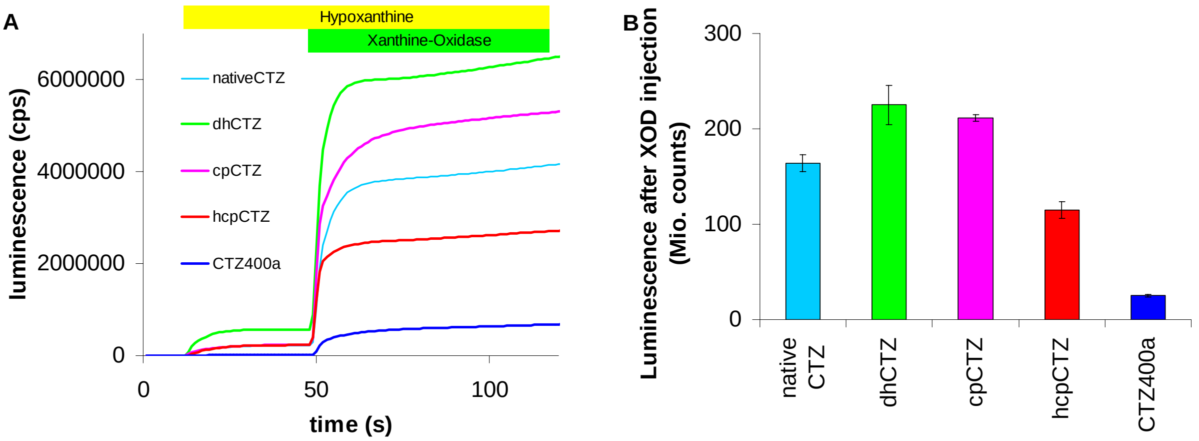

Figure 3.3 The CTZ analogue is crucial for the SOSA assay performance. A: CTZ was mixed with hypoxanthine at t = 12 s. This gives a background luminescence due to the presence of ambient oxygen. The superoxide yielding reaction was started by injection of xanthine oxidase to the assay mix at t = 48 s. **B:** All CTZ analogues apart from CTZ 400a give sufficient light yield when oxidised by superoxide. **C:** The signal-to-background ratio (i.e. the ratio of luminescence in presence of superoxide to luminescence in absence of superoxide) is relevant for the SOSA assay performance. Here CTZ 400a gives the best results. 50 µM CTZ of each analogue was used. Data in B and C are averages of n = 5. Error bars represent StDv.

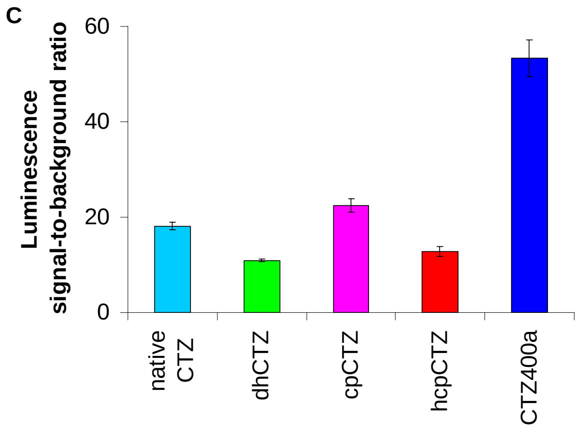

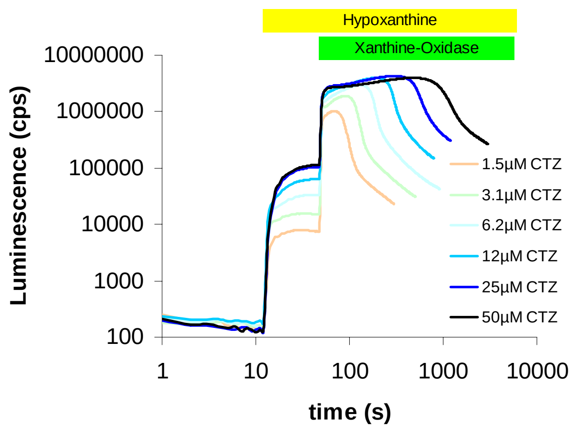

Figure 3.4 The CTZ concentration in the SOSA assay mainly determines the duration of light output, but less the intensity of the superoxide derived luminescence. Each curve represents averages of 3 individual experiments. Note: log-log-scale!

Supplemental References

1. Kervinen M, Pätsi J, Finel M, Hassinen IE: **Lucigenin and coelenterazine as superoxide probes in mitochondrial and bacterial membranes.** *Anal Biochem* 2004, **324**: 45-51.
2. Miller EK, Fridovich I: **A demonstration that O_2^- is a crucial intermediate in the high quantum yield luminescence of luminol.** *J Free Radic Biol Med* 1986, **2**: 107-110.
3. Tarpey MM, White CR, Suarez E, Richardson G, Radi R, Freeman BA: **Chemiluminescent detection of oxidants in vascular tissue - lucigenin but not coelenterazine enhances superoxide formation.** *Circ Res* 1999, **84**: 1203-1211.
4. De Wergifosse B, Dubuisson M, Marchant-Brynaert J, Trouet A, Rees J-F: **Coelenterazine: a two-stage antioxidant in lipid micelles.** *Free Radic Biol Med* 2004, **36**(3): 278-287.
5. Plieth C: **Aequorin as a reporter gene.** *Methods in Molecular Biology* 2006, **323**: 307-327.
6. Teranishi K, Shimomura O: **Coelenterazine analogs as chemiluminescent probe for superoxide anion.** *Anal Biochem* 1997, **249**: 37-43.
