# Supplementary material for: Fingerprinting antioxidative activities in plants
Source: Plant Methods. 2009 Jan 26;5:2. doi: 10.1186/1746-4811-5-2 (PMC2656482; doi:10.1186/1746-4811-5-2)
Supplement: Additional file 4 — The experimental design. The figures summarize the chronology of plant growth, treatment, and harvest and give a scheme how to process the biological material. Fig. 4.1 Experimental design. Fig. 4.2 Flow chart for processing plant material [file 1746-4811-5-2-S4.pdf]

Experimental Design

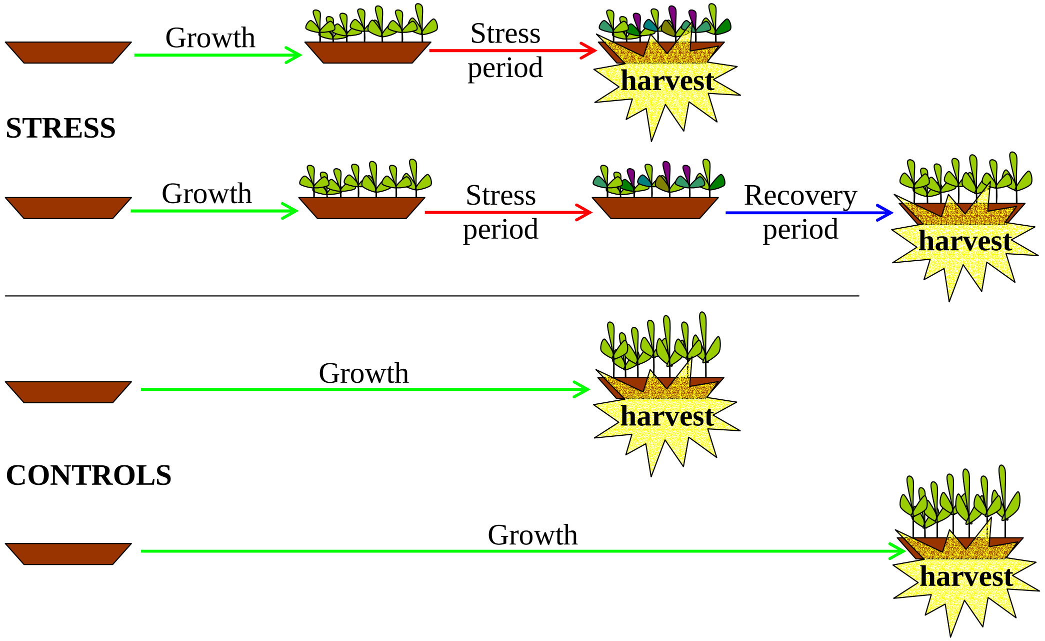

Figure 4.1 Experimental design.
Lepidium sativum seedlings were grown for several days, challenged with abiotic stress and harvested. A part of stressed plants was given a recovery period. Untreated plant material (controls) was always harvested in parallel.

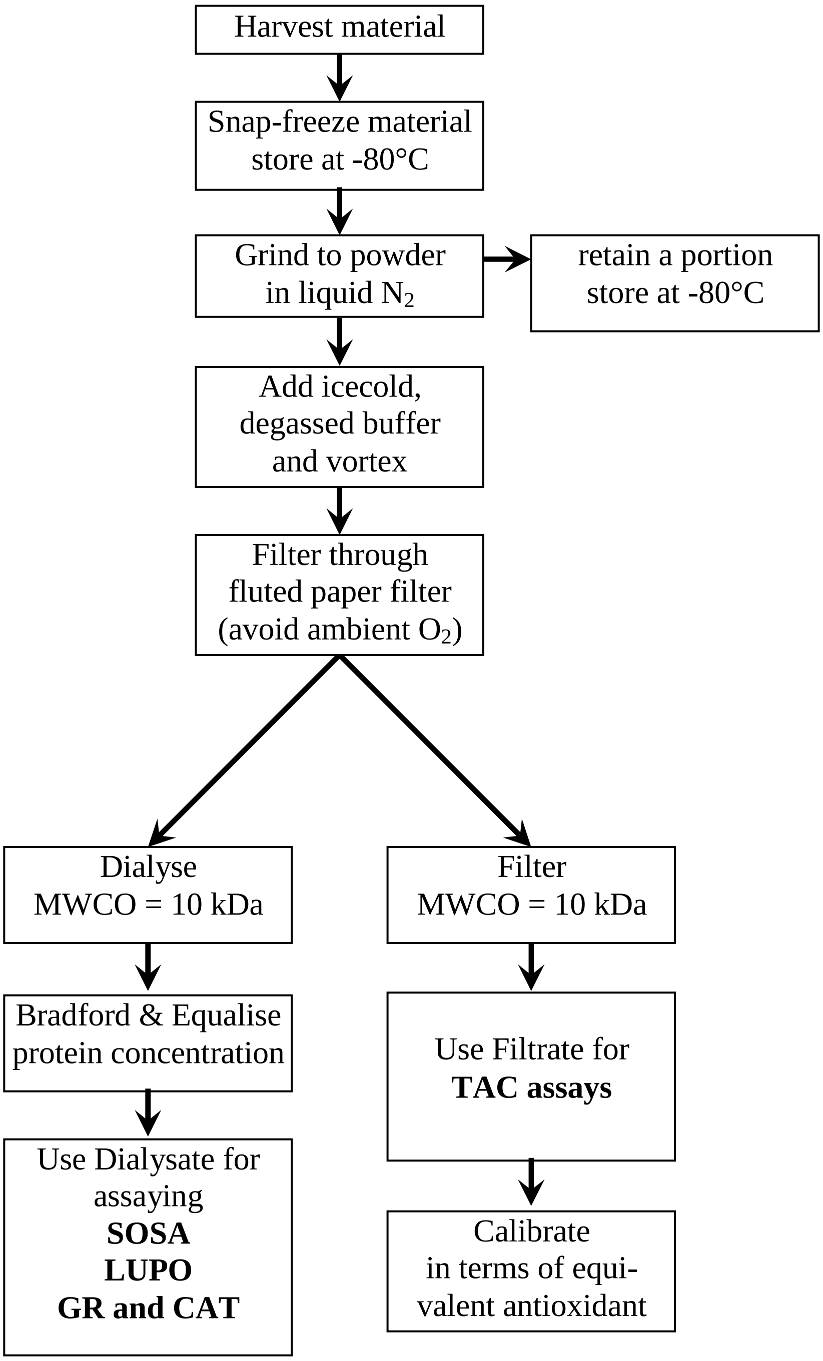

Figure 4.2 Flow chart for processing plant material.
